# Transcriptomic analysis of gene expression in mice treated with troxerutin

**DOI:** 10.1371/journal.pone.0188261

**Published:** 2017-11-30

**Authors:** Yuerong Wang, Shuangshuang Wei, Lintao Chen, Jinli Pei, Hao Wu, Yechun Pei, Yibo Chen, Dayong Wang

**Affiliations:** 1 Hainan Key Laboratories of Sustainable Utilization of Tropical Bioresources, Hainan University, Haikou, Hainan, China; 2 Laboratory of Biotechnology and Molecular Pharmacology, Institute of Tropical Agriculture and Forestry, Hainan University, Haikou, Hainan, China; 3 Department of Animal Science, Hainan University, Haikou, Hainan, China; Defense Threat Reduction Agency, UNITED STATES

## Abstract

Troxerutin, a semi-synthetic derivative of the natural bioflavanoid rutin, has been reported to possess many beneficial effects in human bodies, such as vasoprotection, immune support, anti-inflammation and anti-aging. However, the effects of troxerutin on genome-wide transcription in blood cells are still unknown. In order to find out effects of troxerutin on gene transcription, a high-throughput RNA sequencing was employed to analysis differential gene expression in blood cells consisting of leucocytes, erythrocytes and platelets isolated from the mice received subcutaneous injection of troxerutin. Transcriptome analysis demonstrated that the expression of only fifteen genes was significantly changed by the treatment with troxerutin, among which 5 genes were up-regulated and 10 genes were down-regulated. Bioinformatic analysis of the fifteen differentially expressed genes was made by utilizing the Gene Ontology (GO), and the differential expression induced by troxerutin was further evaluated by real-time quantitative PCR (Q-PCR).

## Introduction

Troxerutin (TX), known as vitamin P4, is a semi-synthetic derivative of the natural bioflavonoid rutin (alias rutoside, vitamin P) which is originally extracted from *Ruta graveolens* L. It is abundant in capers, olive, buckwheat, asparagus, and raspberries[1–4]. Compared with rutin, TX can be absorbed more easily through digestion system. The safety of TX has been confirmed in human including pregnant women and the aged[5–7], clinical trials have proved that TX has a very good safety profile and tolerability even at high doses[8].

Accumulative evidence has shown that TX has broad pharmacological effects, such as vasoprotection, anti-inflammation, immune support and anti-aging[9–16]. It is clinically recognized as an effective agent in treatment of cardiovascular diseases, for instance, varicose veins and chronic venous deficiency[17,18]. It has been reported that TX can pass through the blood-brain barrier and influence the nervous system[19,20]. So far, the effects of TX on genome-wide expression have not been reported, which could help understanding the clinical effects of TX.

In molecular biology, transcript identification and quantification of gene expression has been distinct core activities[21]. The rapid development of high-throughput sequencing of mRNA (RNA-Seq), for example, Illumina RNA sequencing, has provided efficient technology for transcriptomic characterization in human and other well-documented experimental animals like mice[22]. Theoretically, RNA-Seq can characterize every aspect of transcriptional activities. In this context, we used Illumina HiSeq 4000 to generate a substantial dataset of transcript reads in different groups treated with or without TX. To understand the transcriptomic effects of TX, bioinformatics analysis was made which contains Gene Ontology (GO) analyses, and real-time quantitative PCR (Q-PCR) was used to notarize the differential expression of genes induced by TX.

## Materials and methods

### Chemicals

TX was purchased from the Abcam Chemical Company (USA). RNAiso Blood RNA Extraction kit was purchased from Takara, PrimeScript^TM^ RT reagent kit with gDNA Eraser and real-time quantitative PCR kit were also obtained from Takara Bio (Dalian, China). Globin-Zero Gold rRNA Removal Kit for removal of unwanted ribosomal RNA and hemoglobin mRNA from mammalian blood RNA samples were bought from Illumina, Inc. (USA).

### Animal care and maintenance

Male Kunming mice (Swiss mice, SPF), five-weeks old, were purchased from the Experimental Animal Center of Hainan province. They were housed at temperature of 22 to 24 ºC with 12-h light/12-h dark cycle. The mice had free access to water and normal chow diets until they were sacrificed with CO_2_. All efforts were made to alleviate suffering. All animal procedures were abide by the Manipulative Technique for the Care and Use of Laboratory Animals (2nd revision) issued by the State Scientific and Technological Commission of China. All animal experimental protocols were approved by the Institutional Animal Care and Use Committee of Hainan University (Hainan, China). All animals were randomly divided into control group and experimental groups (N = 3). The control group received injection of normal saline (NS), and the experimental group received subcutaneous injection of TX dissolved in NS at dose of 130 mg/kg twice daily at 9:00 AM and 5:00 PM. On the 3th day of treatment, the blood samples were drawn from the animals’ hearts 4 h after the last dose of TX.

### RNA isolation and manipulation

Total RNA was extracted from the blood samples by using a RNA Extraction kit (Takara RNAiso blood). The concentration of RNA in each sample was determined using a NanoDrop 2000 micro-volume spectrophotometer (Thermo Scientific, USA), and the quality of RNA samples were examined using an Agilent Bioanalyzer (Agilent Technologies, USA). The defined criterion for qualification of RNAseq is that the RIN value of a sample is higher than 8.5. Ribosomal RNA and hemoglobin mRNA from the blood RNA samples was removed using a Globin-Zero Gold rRNA Removal Kit (Illumina, USA).

### Sequencing library construction and illumina sequencing for transcriptomic analysis

The sequencing library construction and Illumina sequencing were performed by AnnoRoad Biotechnology Co, Ltd, China. Briefly, mRNA was enriched via purification with oligo (dT) magnetic beads and fragmented into short fragments using fragmentation buffer. First-strand cDNA was synthesized using random primers and fragments of mRNA as templates. Subsequently, second-strand cDNA was synthesized using DNA polymerase I, buffer, dNTPs, and RNaseH. After purification, we used a QIAQuick PCR kit and eluted by EB buffer. Purified double-stranded cDNA were end-repaired, added bases A, and ligated with sequencing adaptors. After SDS-PAGE, amplified products were gel extracted. The cDNA were PCR-amplified for a minimum number of cycles to avoid normalization for downstream quantitative gene expression analyses. Then it was sequenced on an Illumina HiSeq 4000 with the sequencing strategy PE150.

### Reads processing and identification of differentially expressed genes

To ensure reproducibility and reliability of the results, quality control checks were applied pertinently at different stages of the analysis. Quality control for the raw reads contained the analysis of sequence quality, GC content, the presence of adaptors and low-quality reads. We adopted DESeq to analyze differential expression of genes (DEGs). Compared with the reference genome of Kunming mice, we selected standard was |log2Ratio|≥1 and q<0.05, then got up-regulated and down-regulated genes. All expressed genes were monitored, and their gene functions were explored using database annotations such as NR, NT, UNIPROT, GO, and KEGG.

### Real-time quantitative PCR

Reverse transcription was carried out using PrimeScript^TM^ RT reagent kit with gDNA eraser (Takara, China). Total RNA was isolated from the blood samples drawn from mice hearts. For cDNA synthesis, one μg of total RNA was reverse-transcribed in a total volume of 20 μL, using a One-step gDNA Removal and cDNA Synthesis Super Mix kit (Trans Script).

Synthesized cDNA samples were diluted 3 times prior to Q-PCR. The primers were designed for 9 genes (Table 1). Q-PCR was accomplished using the SYBR premix Ex Taq kit (Takara, China). β-actin was used as the internal reference gene in this study. The results were analyzed on an ABI StepOnePlus Real-Time PCR System (Thermofisher Scientific, USA).

**Table 1:** Q-PCR primers.

Target gene	Primer sequence(5’ to 3’)		Product length (bp)
β-actin	Forward	GGCTGTATTCCCCTCCATCG	154
	Reverse	CCAGTTGGTAACAATGCCATGT	
Tnr	Forward	GGAGGTGACTACAGAAAGGGC	137
	Reverse	AGAGGCTTTCAAGTGGCACG	
Robo1	Forward	TCCAAAGAGAACTGGGGAATGT	142
	Reverse	GCTCCAGATGGGCGGTAG	
Pnp2	Forward	CGACCTCAAGTGGCAGTGAT	148
	Reverse	GCAATCCAAACACCAGTCGG	
H2-k1	Forward	TGGACGACACGGAGTTCG	147
	Reverse	CCACTCGGAAACTCTGCTCAT	
Fcnb	Forward	CTGACTGTCCATGCGGCTG	147
	Reverse	TCCTCTATCTCCTTTGGCACC	
Arhgdig	Forward	GCTTGGTCAAGTACAAGCAGG	126
	Reverse	CCATGATGATAGGCCCTGGAG	
Hbq1a	Forward	CGGAATCTACACGACCGAGG	146
	Reverse	TAGCGAGAGTCAGTGCATCG	
Srp14	Forward	CGTGTTCATCACCCTCAAGAAAT	136
	Reverse	CACGGTGCTGATCTTCCTTTT	
Npy	Forward	GGCCAGATACTACTCCGCTC	135
	Reverse	CTTGTTCTGGGGGCGTTTTC	

Q-PCR was performed with 2 μl of template cDNA, 0.8 μl of forward primer (10 μM), 0.8 μl of reverse primer (10 μM), and 10 μl of SYBR Primer Ex Taq II (Tli RNaseH Plus) (2 X) in a total reaction volume of 20 μl. The procedure was conducted as follows—95°C for 30 s for initial denaturation followed by 40 cycles of 95°C for 5 s, and 60°C for 34 s—and then generated the melt curves for verification of amplification specificity by a thermal denaturing step. Genes was normalized to β-actin expression and calculated using the equation: change (x-fold) = 2^-ΔΔCt^[23].

Experiment data were analyzed using IBM SPSS statistics 19 software. Statistical significance was determined by the Student’s t test or one-way analysis of variance (ANOVA), and the differences were considered statistically significant at a value of P < 0.05.

## Results

### Illumina sequencing and data quality

Initial llumina sequencing results existed in the original image data file. Using the CASAVA softwarh the base calling function, they were transformed into sequenced reads, which were called the raw data. The raw data were stored in FASTQ file format. FASTQ files include the name of every read, base sequence and their sequencing quality information.

The raw reads output from the Illumina sequencing platform were trimmed for the purpose of quality control. Adaptor, N< 10% and low-quality reads were removed to obtain high-quality reads, which was called clean reads, producing about 23.76 million paired-end reads per sample equivalent to over 3.405 to 3.564 billion nucleotides per sample. Among those reads, over 96.27% of the clean reads with high-quality scores at the Q30 level (a base quality greater than 30 and an error probability of 0.1%) were identified (Table 2).

**Table 2:** Summary of statistical data for the transcriptome of mice.

Samples	C1	C2	C3	YR1	YR2	YR3
**Total raw reads**	25,037,206	24,771,616	23,903,170	24,938,022	25,995,740	24,499,022
**Total clean reads**	23,764,936	23,558,544	22,702,076	23,519,160	23,737,328	23,042,794
**Clean bases Number**	3,564,740,400	3,533,781,600	3,405,311,400	3,527,874,000	3,560,599,200	3,456,419,100
**Clean reads rate (%)**	94.92	95.1	94.98	94.31	91.31	94.06
**Clean Q30 bases rate (%)**	98.04	98.03	98.11	97.58	96.27	97.52

**Fig 1:**
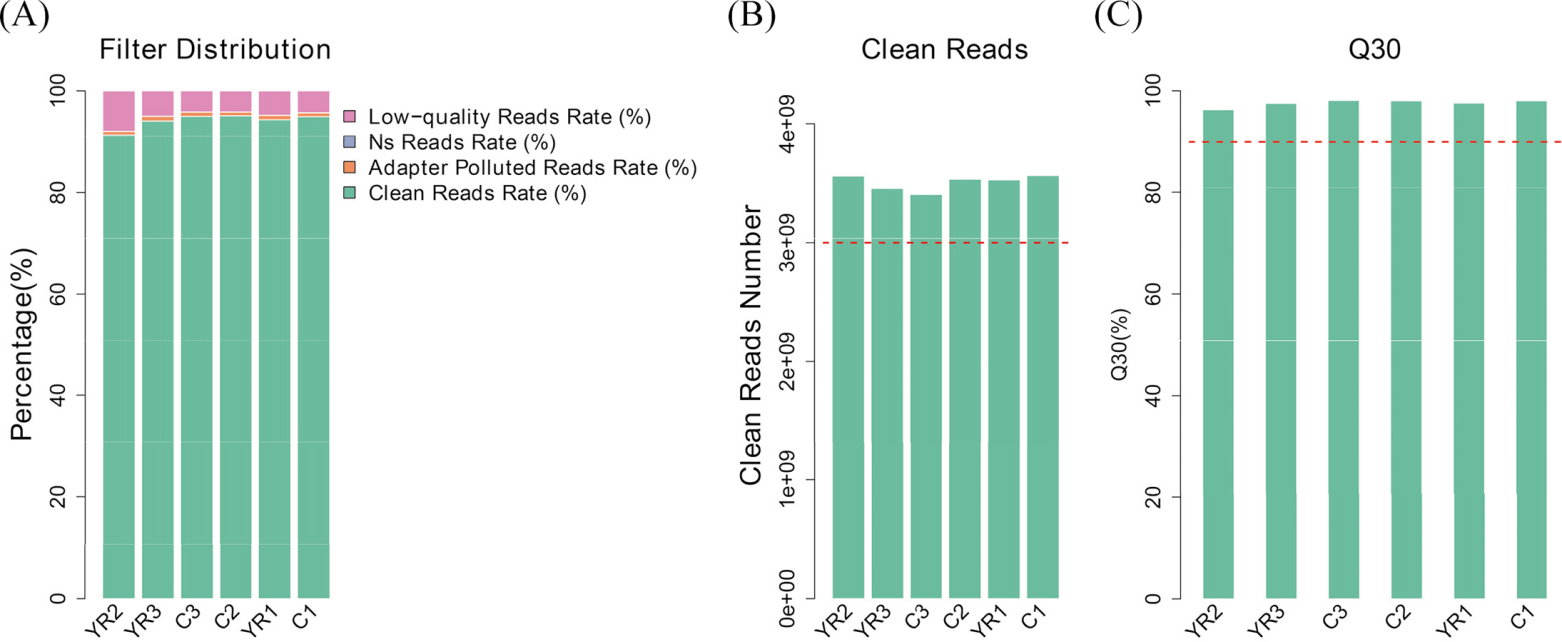
**A. Distribution of reads proportion before filtering.** C1, C2 and C3 represent control groups that received injection of normal saline (NS), and YR1, YR2 and YR3 represent experimental groups that received subcutaneous injection of TX dissolved in NS at a dose of 130 mg/kg, twice daily. **B. Data volumes of clean reads for samples.** For the purpose of quality control, the raw reads output from the Illumina sequencing platform were filtered: adaptors, short reads with N < 10%, and low-quality reads were removed. **C. Q30 proportion for samples.** The clean reads with high-quality scores at Q30 levels (a base quality greater than 30 and an error probability less than 0.1%) are more than 95%. With a vast majority of bases scoring no less than the Q30 level, the levels of accuracy are ideal for sequencing applications.

**Fig 2:**
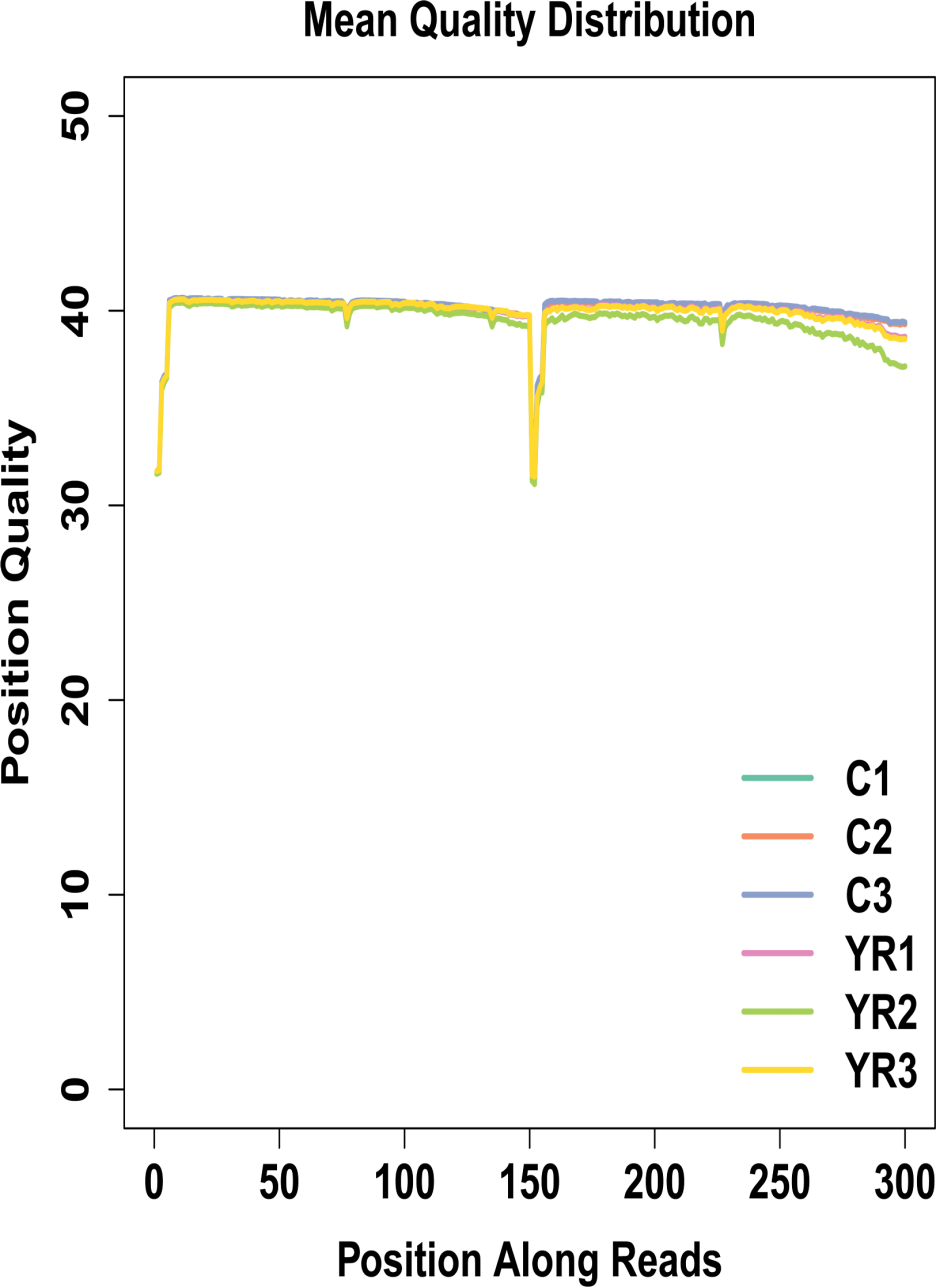
Mean quality distribution of samples. C1, C2 and C3 represent control groups that received injection of normal saline (NS), and YR1, YR2 and YR3 represent experimental groups that received subcutaneous injection of TX dissolved in NS at a dose of 130 mg/kg, twice daily. The x-axis indicates base position of filtered high quality sequences. The y-axis indicates mean sequencing quality at each position.

The clean reads were used for further analysis (Fig 1A and 1B). The cDNA libraries produced a total of 140,324,838 clean reads, which represented the majority of the data (almost covered 90% of the whole gene set), with Q30 scores > 95% (Fig 1C). The mean quality distribution of all samples was shown in Fig 2.

### Comparison analysis with a reference genome and gene mapping

**Fig 3:**
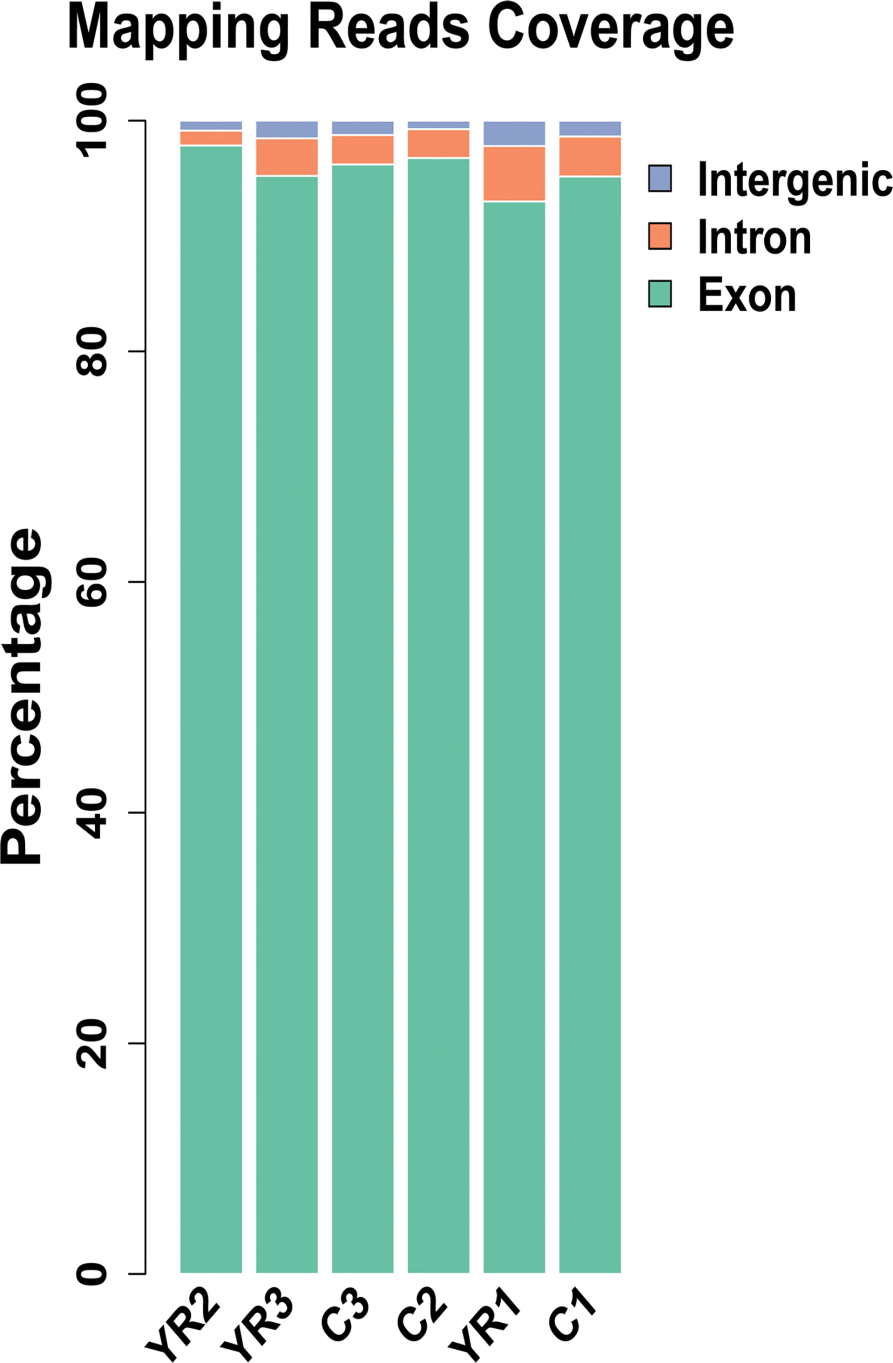
Reads coverage mapping to the reference genome of Kunming (Swiss) mice. C1, C2 and C3 represent control groups that received injection of normal saline (NS), and YR1, YR2 and YR3 represent experimental groups that received subcutaneous injection of TX dissolved in NS at a dose of 130 mg/kg, twice daily. Blue, green and orange refer to intergenic, exon and intron region respectively.

The filtered sequencing results were compared with the reference genome of Kunming mice for its orientation to the genome. For the samples, we used TopHat software, which was specifically designed for comparison of the transcriptome data[24,25]. To ensure accuracy and reliability of the data, about 140 million high-quality reads were generated, and over 88.5% of the data were mapped to the reference genome for each sample. Most of the sequences were compared to the exon region. Besides, alternative splicing and expression noise maybe come from the intron region, and the new transcripts and expression noise may belong to the intergenic sequence. For the control group and experiment group, 95.35% and 96.04% were mapping to the exon region, 3.12% and 2.84% belonged to the intron region, and 1.53% and 1.12% were in the intergenic sequence respectively (Fig 3) (Table 3).

**Table 3:** Summary statistics of clean reads mapping.

Sample	C1	C2	C3	YR1	YR2	YR3
**Total Clean Reads**	23,764,936	23,558,544	22,702,076	23,519,160	23,737,328	23,042,794
**Mapping Reads**	22,158,309	22,118,461	21,098,768	20,836,785	21,276,557	20,227,093
**Mapped Rate (%)**	0.93	0.94	0.93	0.89	0.9	0.88
**Exon (%)**	95.16%	96.76%	96.20%	92.99%	97.84%	95.21%
**Intron (%)**	3.47%	2.50%	2.55%	4.81%	1.30%	3.26%
**Intergenic** **(%)**	1.37%	0.74%	1.25%	2.20%	0.86%	1.54%

### Analysis of differential expression of genes

Differential expression analysis requires that gene expression values be compared among samples. Reads Per Kilobase Million (RPKM) is used to quantitatively estimate value of gene expression[26], which has the computation formula:

RPKM=(1000000*R)/(N*L/1000)

Where R is the number of mappable reads in specific genes, N is the total number of reads mapped to genes in a particular sample, and L is the length of the gene exon.

In general, differential gene expression only account for a small portion of total gene. There is little effect on the distribution of the sample expression quantity by a few differentially expressed genes. All samples have similar overall gene expression pattern (S1 Fig).

Differences in gene expression in mice in two different groups were examined. For biological triplicate samples, we adopted DESeq to analyze differential expression of genes. In total, fifteen genes were differentially expressed, among which 5 genes were significantly up-regulated and 10 genes down-regulated in TX-treatment mice (S2 Fig). We identified their gene functions using database annotations tools such as NCBI, UNIPROT, and GO databases. We summarized 15 different genes main function influenced by TX in mice (Table 4).

**Table 4:** Summary of 15 genes for the transcriptome of mice.

Gene	variation tendency	Main function
*Tnr*	up	cure neurological diseases and neuroprotection
*Hbq1a*	up	impact the activity of oxygen transporter, anti-oxidative
*RPL17e*	up	important for ribosome architecture and function, tumor suppressor
*GM10499*	up	against extracellular microbes and toxic molecules
*GM5526*	up	responsible for skeletal muscle tissue development and muscle filament sliding
*Robo1*	Down	protect liver from injury and fibrosis
*Pnp2*	Down	positively regulate T cell-mediated cytotoxicity and proliferation of T cells
*H2-k1*	Down	anti-inflammatory and immune
*Fcnb*	Down	ameliorate inflammation
*Arhgdig*	Down	catalytic activity and protein localization
*Srp14*	Down	protein export
*Npy*	Down	immunomodulatory function, maintenance of body homeostasis and inhibits diabetes
*Rpl13e*	Down	drug resistance, virus replication and anti-oxidative defense
*GM28114*	Down	against extracellular microbes and toxic molecules
*GM12366*	Down	anti-cancer

### Gene Ontology (GO) analysis for selected gene

**Fig 4:**
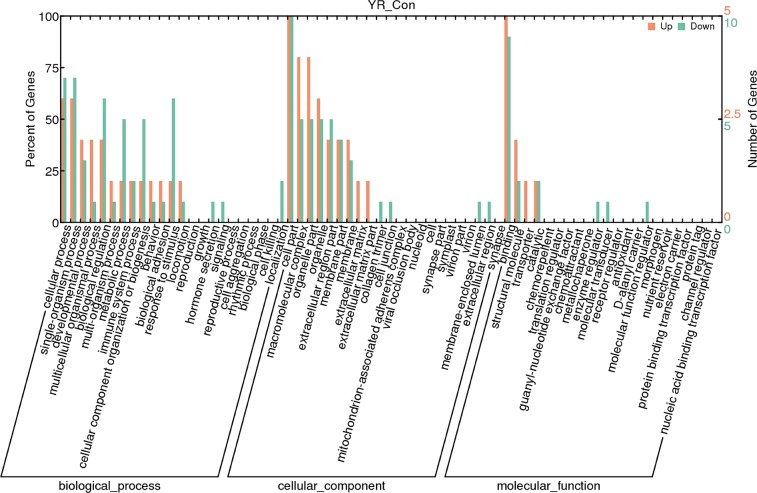
Gene Ontology (GO) classification of differentially expressed genes (DEGs). DEGs are classified into three major domains: biological process (BP), cellular component (CC) and molecular function (MF). The left y-axis indicates the percentage of a specific category of genes in a domain. The right y-axis indicates the number of genes in the category. (Con, control group; YR, experimental group).

To further understand the function of the differential expression of genes, GO term enrichment analysis (q<0.05) was performed. There are three GO domains: biological process (GOBP), cellular component (GOCC), and molecular function (GOMF). The most significantly enriched GO terms were “single-organism process” and “biological regulation” within GOBP, ‘binding’ and ‘structural molecule’ were the two most abundant terms within GOMF, and “macromolecular complex” and “organelle part” were the most highly represented terms in GOCC respectively (Fig 4).

A directed acyclic graph (DAG) showed enrichment analysis results. It is a branch inclusion relationship that the lower node function was subordinate to the upper nodes function. Top 5 GO enrichment analysis results were selected as master nodes in DAG. The color depth of the DAG nodes indicates the degree of enrichment, the darker the red color is, the higher the enrichment degree of the node would be.

**Fig 5:**
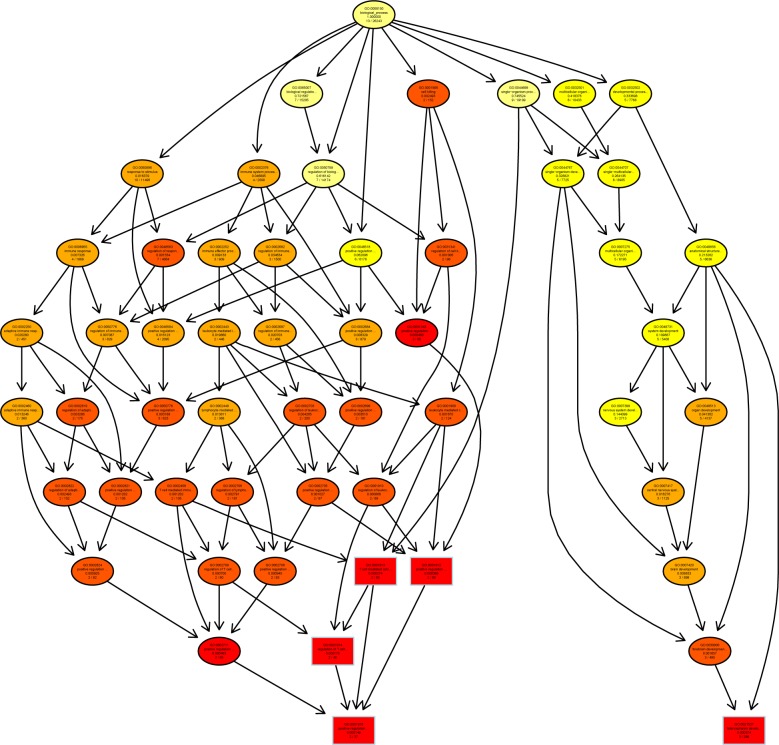
Directed acyclic graph (DAG) for differentially expressed genes (DEGs) in the biological process (BP) ontology.

**Fig 6:**
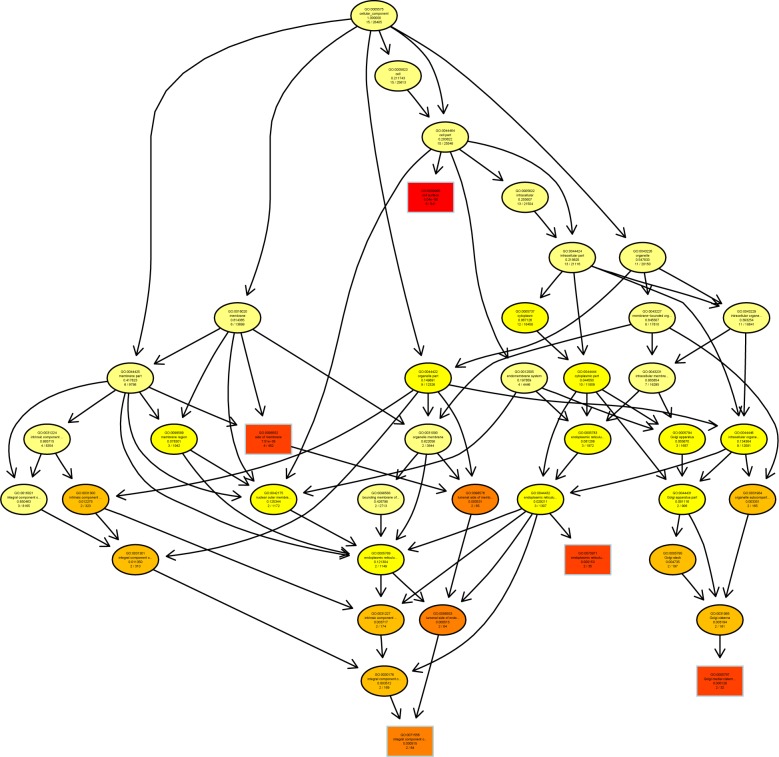
Directed acyclic graph (DAG) for differentially expressed genes (DEGs) in the cellular component (CC) ontology.

**Fig 7:**
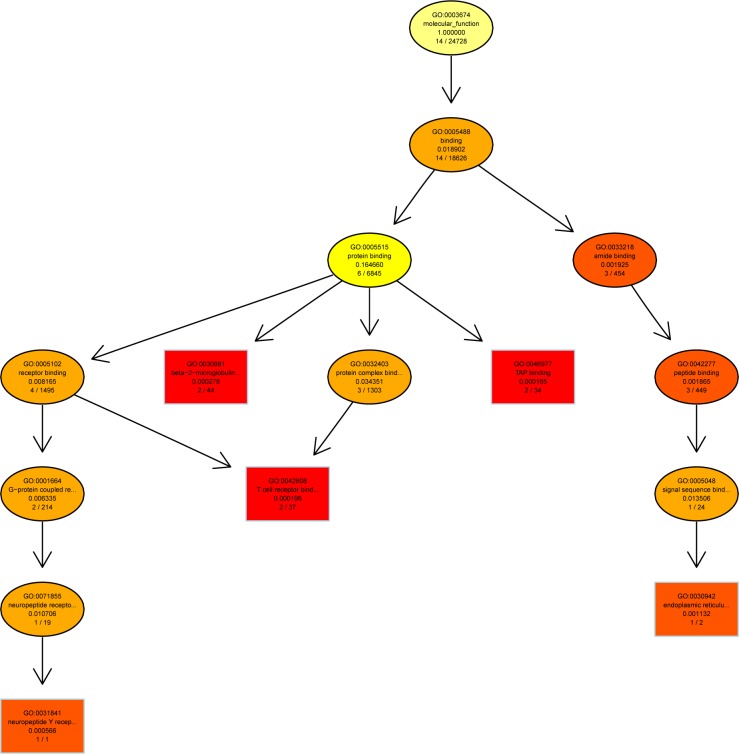
Directed acyclic graph (DAG) for differentially expressed genes (DEGs) in the molecular function (MF) ontology.

We found that genes involved in T cell mediated cytotoxicity (q = 0.000149) and telencephalon development (q = 0.000374) were most highly enriched (Fig 5). Further characterization of the differential expression of genes using DAG showed that the differentially expressed genes were implicated in cell surface, cell membrane (q = 5.04e-06) and endoplasmic reticulum exports (q = 0.000153) (Fig 6). In molecular function, genes in β2-mioroglobule binding (q = 0.000278), TAP-binding (q = 0.000165), T cell and neuropeptide Y receptor binding (q = 0.000196) were highly enriched (Fig 7).

### Real-time quantitative PCR

**Fig 8:**
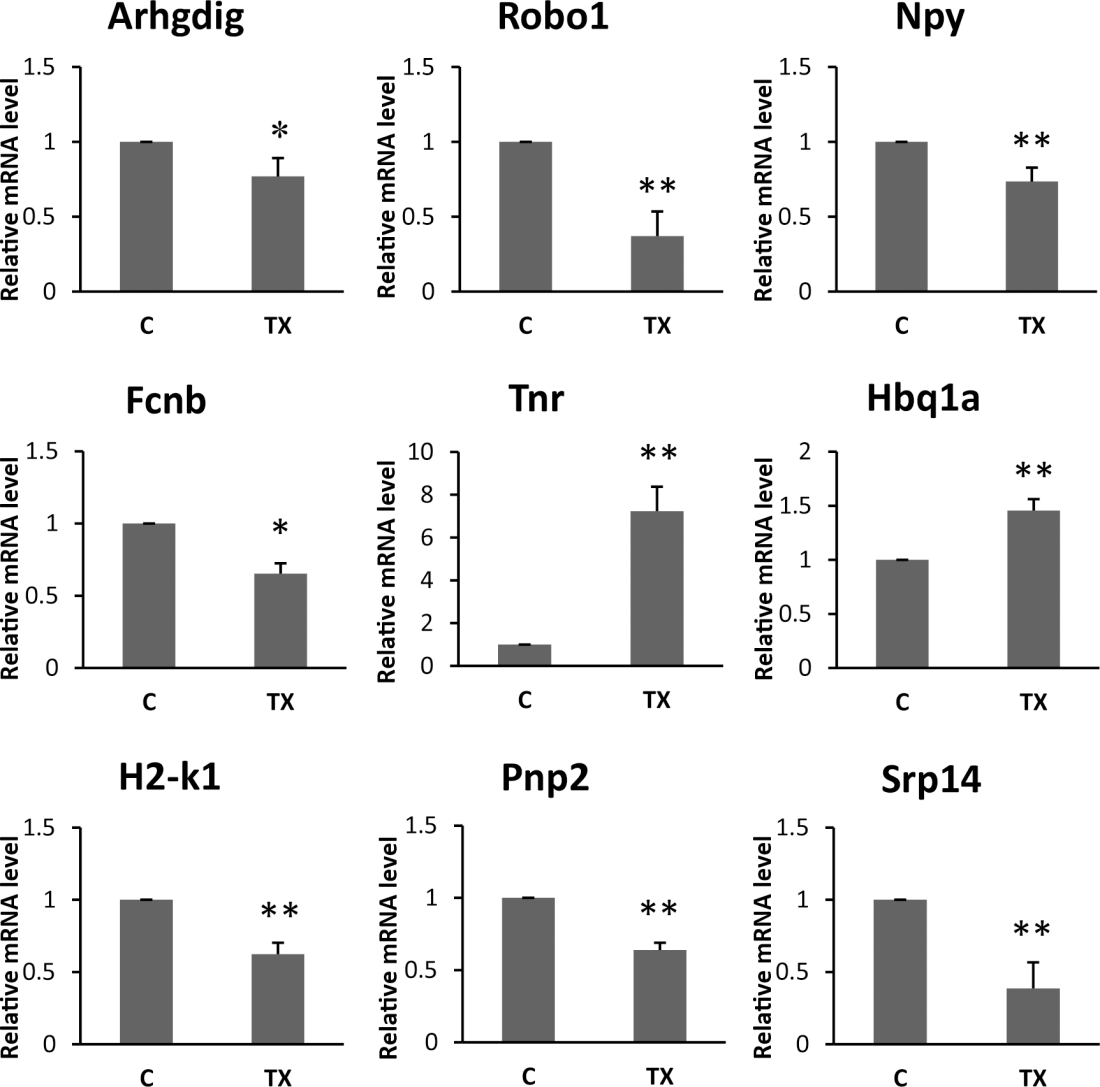
Q-PCR for nine differentially expressed genes (DEGs). Relative gene expression was normalized by comparison with the expression of β-actin, and calculated using the 2^−ΔΔCT^. (C, control group; TX, troxerutin, s.c. injected at a dose of 130 mg/kg, twice daily. *P < 0.05 and ** P<0.01 vs control group, N = 6–8).

**Fig 9:**
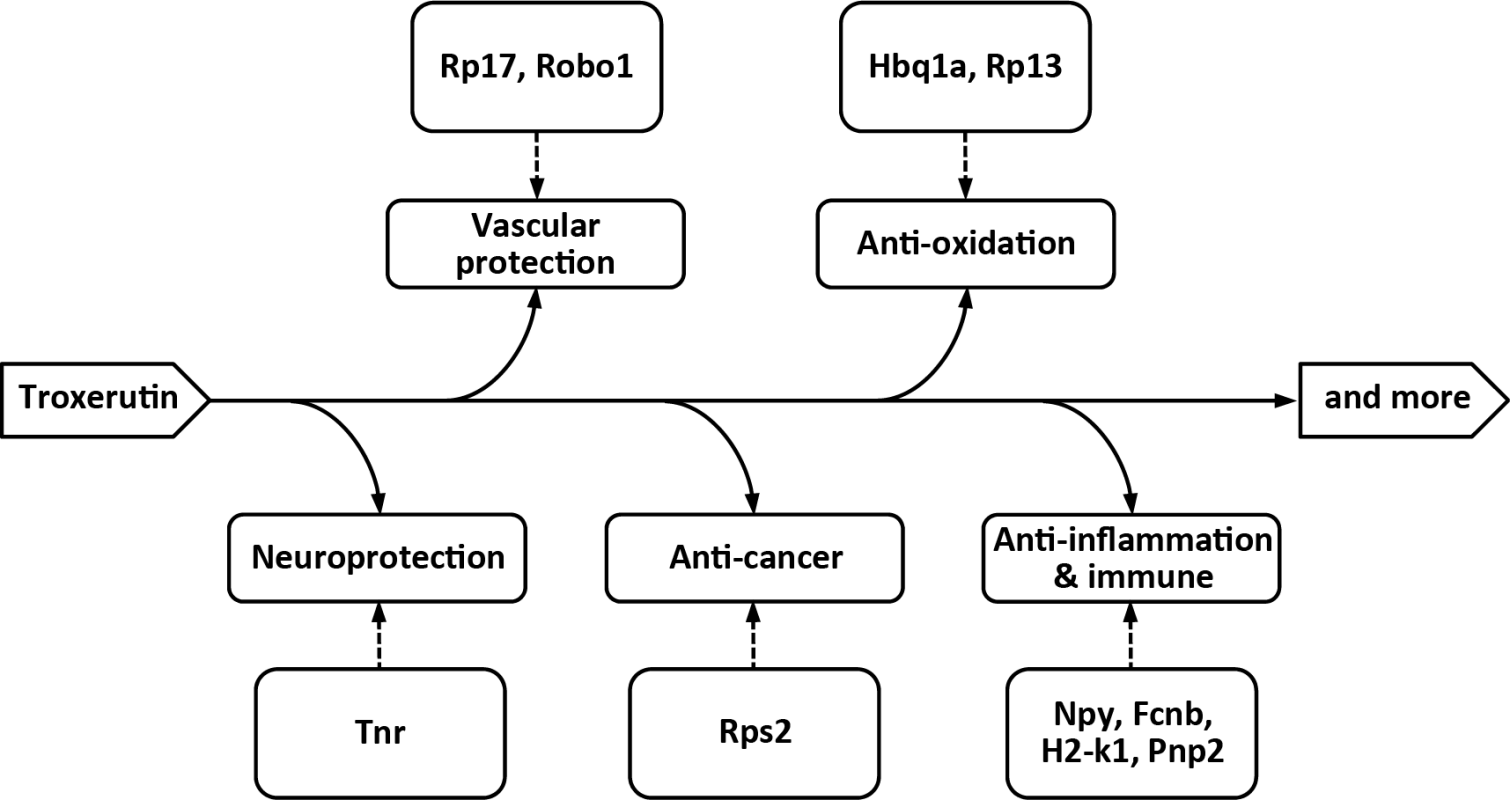
Pleiotropic effects of troxerutin.

To further confirm the accuracy and reproducibility of the RNA-Seq results, real-time quantitative Q-PCR was performed to quantify the transcriptional levels. Nine differentially expressed genes, including *Tnr*, *Robo1*, *Npy*, *Arhgdig*, *Srp14*, *H2-k1*, *Pnp2*, and *Fcnb*, *Hbq1a* were evaluated, and each genes showed significantly differential expression after the treatment with TX (Table 5) (P<0.05, Fig 8). We performed the transcriptomic characterization of the effects of TX in mice to identify differentially expressed genes related with pharmacological properties of TX (Fig 9).

**Table 5:** Relative expression values determined using RNAseq and real-time quantitative PCR (Q-PCR).

gene	RNA-seq		Q-PCR(±SD)	variation tendency	
	Fold Change	Log2Fold Change	Relative expression values(2^−ΔΔCT^)	RNA-seq	Q-PCR
*Tnr*	187.9821351	7.554451752	6.7992±1.27	Up	Up
*Robo1*	0.005668298	-7.462868797	0.3707±0.11	Down	Down
*Pnp2*	0.024258529	-5.365364134	0.5447±0.09	Down	Down
*H2-k1*	0.127654812	-2.969680168	0.7192±0.07	Down	Down
*Fcnb*	0.036825424	-4.763154068	0.5041±0.04	Down	Down
*Arhgdig*	0.015302976	-6.030043964	0.7694±0.12	Down	Down
*Hbq1a*	298.7735665	8.222908703	1.4009±0.15	Up	Up
*Srp14*	0.199374235	-2.326449115	0.4807±0.10	Down	Down
*Npy*	0.000831662	-10.23171562	0.3677±0.01	Down	Down

## Discussion

TX, a rutoside derivative, has raised considerable interest because of its extensive pharmacological activities. It has been conventionally used for treating diseases including capillary fragility, chronic venous and varicosity in clinical[27]. Researches over the past decades have revealed that TX can act againstγ-radiation-induced lipid peroxidation, UV radiation and colon carcinogenesis[28–30]. Furthermore, TX can cure retinopathy and play a significant role in the management of type-2 diabetes mellitus[31–33]. Beside, TX can inhibit 2-AA + UVA radiation-induced DNA damage and environmental carcinogens, showing its potential therapeutic activities for cancer[34]. Gene expression behind the pharmacological activities of TX has not been investigated clearly so far. And our findings provide novel insights into TX, which could help understanding clinical effects of TX.

In this study, we made a comprehensive analysis of the effects of TX on genome-wide expression of genes. A total of 15 differentially expressed genes (DEGs) were found, in which 5 genes were up-regulated and 10 genes down-regulated significantly. Moreover, Q-PCR confirmed that our RNA-Seq results were reliable. Our experimental findings provide evidence about the TX against diabetes, cardiovascular diseases and cancer through down- or up-regulation of different genes, implicating its treating capacity.

Tenascin-R (TN-R) is an extracellular matrix protein expressed primarily in the central nervous system. It is a member of the tenascin gene family, which includes 4 known members in vertebrates. During development of mammalian brain, TN-R is found around birth[35]. TN-R can affect cell migration, cell differentiation and adhesion. TN-R expression is tightly regulated in a spatiotemporal manner in brain, especially during cortical plate formation[36]. More importantly, many researches showed that TN-R could play an important role in brain development and cognition. By activating microglia, TN-R can indirectly elevate the secretion of cytokines and growth factors, including brain-derived neurotrophic factor (BDNF), transforming growth factor-β (TGF-β), CXCL2, and nerve growth factor (NGF)[37]. In addition, loss of TN-R was shown to impair cognition, synaptic plasticity and motor abilities[38]. And it has been proved to participate in maintaining a balance between excitatory and inhibitory circuits involved in learning, memory and cognition in mice[39–41]. TN-R deficient mice showed increased excitatory transmission through reduced perineuronal nets and altered synaptic activities[42]. In summary, TN-R may play a primary or secondary role in many neurological diseases [43] and have a significant role in neuroprotection. Previous evidence showed that TX significantly improved behavioral performance of D-galactose-treated mice in the step-through test and Morris water maze[44], and alleviated the oxidative damage caused by D-galactose in the liver and kidney, as well as cognitive impairments. In addition, some researchers think TX could be recommended as a possible candidate for prevention and therapy of cognitive deficits and Alzheimer's disease[45], and improved synaptic plasticity failure induced by Amyloid β peptide. The up-regulation of *TN-R* expression induced by TX may underlie its neuroprotective function.

Neuropeptide Y (NPY), a 36-amino acid peptide [46], is a highly conserved neurotransmitter involved in a broad range of fundamental physiological activities. NPY’s pleiotropic functions comprise regulation of body-weight and energy balance, brain activity, food intake, vasoconstriction, immune function, and emotional response[47]. In fact, its immunomodulatory function and maintenance of body homeostasis are also vital [48]. The effects of NPY are depend on many factors, such as at least four G protein-coupled receptors known as Y1, Y2, Y4, Y5 and cell types involved[49]. Different receptors are involved in various physiological activities. During inflammation, immune cells themselves are capable of producing and releasing NPY. NPY can regulate immune cell activities through a paracrine or autocrine mode of action[50–52]. NPY has different effects on inflammation. The binding of NPY to receptors influences the activities of immune cells in either a pro-or an anti-inflammatory manner[53]. Recently, it has been proved that NPY possesses a pro-inflammatory function in the gut[54,55]. Additionally, loss of NPY can combat obesity and diabetes through increased energy expenditure and lowered fat contents. In our study, we found that under the effect of TX, the *NPY* expression was suppressed. It provides evidence that TX can ameliorate lipid abnormalities and diabetes[56,57], and have anti-inflammatory activities at least in part.

Ribosomes are essential components of the protein synthesis machinery. Ribosomes are formed from two unequally sized subunits: the large ribosomal subunit and small ribosomal subunit[58]. The process of ribosome biogenesis is well organized and tightly regulated. There is increasing evidence indicating that ribosomal proteins (RPs) have extraribosomal functions including DNA repair and other cellular processes. It has been shown that RPs are related to the development and progression of metabolic, cancer and cardiovascular diseases [59]. 60S ribosomal protein L17 (rpl17) is a protein that is encoded by the *Rpl17* gene. It has an essential role in ribosome architecture and function. Rpl17 is thought to stabilize long-range interactions important for establishing the structure of the polypeptide exit tunnel, during 60S subunit assembly[60]. Reducing the amount of rpl17 in mouse cells led to stalled 60S subunit maturation, causing degradation of most of synthesized precursors, which could affect normal functions of the ribosome. Elaine et al. discovered that rpl17 is a vascular smooth muscle cell (VSMC) growth inhibitor, akin to a tumor suppressor[61]. In our study, *Rpl17* was found to be up-regulated by TX. It could have relationship with that TX cures cardiovascular diseases and is conducive to the formation of ribosomes. Besides, ribosomal protein L3 (rpl3) is known to be an essential component for the peptidyltransferase center[62]. It acts as a binding site for a ribosome inhibitory protein and plays an important role in drug resistance and virus replication[63]. Annapina et al. revealed that *Rpl3* was down-regulated and the expression of stress-response genes was activated in rCalu-6 cells. They found that rpl3 reduced xCT and GST-a1 expression levels. Researches also demonstrated that rpl3 was a negative regulator of cystathionine-β-synthase (CBS) expression. Silencing CBS gene severely reduces cellular glutathione (GSH) levels, and down-regulation of Rpl3 can induce anti-oxidative defense. These represent indirect ways for TX to potentiate expression of antioxidant genes to activate defensive response. Rpl3 also regulates negatively the activation of NF-kB[64]. Rps2, a 32 kDa ribosomal protein, is over expressed in prostate cancer and promotes malignancy of human prostate PC-3ML cells in the severe combined immunodeficiency (SCID) tumor modeling studies[65]. Previous studies have found that *Rps2* is overexpressed in human squamous cell carcinoma and breast tumor samples. Thus, rps2 might promote cancer and be an excellent therapeutic target for the treatment of the diseases. In this context, the effect of TX to inhibit *RpS2* expression and thereby stabilize it in cells indicates that TX may have anti-cancer function.

The mammalian signal recognition particle (SRP) is an 11S cytoplasmic ribonucleoprotein that plays an essential role in protein sorting. It is a cytosolic particle that transiently binds to the endoplasmic reticulum (ER) signal sequence of a nascent protein, to the large ribosomal unit, and to the SRP receptor in the ER membrane[66]. *Srp14* encodes SRP subunit 14 (SRP14). It can influence protein export, as specified in the Gene Ontology Molecular Function (GOMF). SRP14 and SRP9 proteins form a heterodimer that bind to the RNA of Alu domain of SRP which is responsible for translation arrest. This gene is significantly down-regulated by TX. Besides, GTP hydrolysis plays a vital role in the SRP cycle; SRP protein and both subunits of the SRP receptor contain G-domains. The GTPase cycle of SRP, modulated by the ribosome, provides the regulatory link between translation and translocation machineries[67,68]. Interestingly, in our study, *Arhgdig* was found to be down-regulation by TX. It’s Gene Ontology biological process (GOBP) is the regulation of catalytic activity and protein localization. This gene encodes a protein which is a GTPase activator. To sum up, our study indicated that that TX may have pharmacological effects on the synthesis and export of proteins.

Form the DAG, we found that the genes involved in T cell-mediated cytotoxicity and telencephalon development were highly enriched. T cells help eliminate pathogens present in infected cells and also help B cells make different kinds of antibodies to protect against extracellular microbes and toxic molecules[69]. To accomplish these important functions, T cells have to interact intimately with other cells and then come into play. However, T cells are unable to peek beneath the surface of cells to identify the cells that have ingested bacteria. T cells recognize antigenic peptide only in the context of MHC I or MHC II molecules that are displaying the antigen on cell surface[70]. MHC I molecules present peptides from the proteins that are synthesized by cells. This recognition allows the immune system to detect cells in abnormal states for elimination. Moreover, Reduction of MHC I molecule expression in human tumors is often detected[71,72]. Among DEGs, *H2-k1* and *GM10499* gene encode major histocompatibility complex class I (MHC-I), and *GM28114* encodes lymphocyte antigen 6 complexes. Expression of these genes are differently affected by TX. In GOBP, they can positively regulate T cell-mediated cytotoxicity and endocytosis. Besides, PNP2 is a family of purine-nucleoside phosphorylase. It can also positively regulate T cell-mediated cytotoxicity and proliferation of T cells. *PNP2* expression is down-regulated by TX.

Natural immunity depends on the ability of some pattern recognition molecules to sense molecular markers. Ficolin is a group of pattern recognition molecules to recognize molecules on pathogens, apoptotic and necrotic cells. A series of new findings have indicated that ficolin B encoded by *Fcnb* gene can activate the complement system via the lectin pathway similar to MBL[73–75]. The activation of the complement system results in the release of multiple inflammatory signaling molecules[76]. Recently, a number of reports have shown that membrane attack complexes (MACs) were produced by complement activation. MACs can increase production of pro-inflammatory cytokines IL-8 and NF-kB nuclear translocation. These cytokines and molecules can induce inflammation. Moreover, Ficolins are reported to stimulate expression of inflammatory cytokines by macrophages[77]. Under the effect of TX, the expression of *Fcnb* gene was down-regulated, which may ameliorate inflammation.

The Roundabout (Robo) family proteins are transmembrane receptors. Secreted proteins Slit-family proteins (SLITs) can act through Robo receptors to mediate axonal guidance and branching. It could be related with directing migration of many cell types, such as immune, and tumor cells[78]. Robo1 was initially found in Drosophila. SLIT/ROBO signaling is an important regulator of cellular interactions[79]. Some researchers have proved that Slit2-Robo1 signaling may debilitate liver injury and fibrosis[80]. In our study, Robo1 gene expression was decreased by TX, which could protect liver from injury and fibrosis. In addition, Lvzhen et al. demonstrated that silencing the expression of *Robo1*gene inhibited cell proliferation and suppressed the development of proliferative vitreoretinopathy (PVR), offering a potential therapeutic usefulness in treating PVR[81]. Other studies support that ovarian angiogenesis was enhanced by a partial lack of Robo1 genes and this lack would enhance ability of fertility[82].

In addition to the above, *Hbq1a* encodes hemoglobin, theta 1A, which is closely connected with oxygen and iron binding, and impact the activity of oxygen transporter. It may be associated with anti-oxidative effects of TX[83]. *GM5526* is mainly responsible for skeletal muscle tissue development and muscle filament sliding. It encodes a protein, which is a structural constituent of muscle.

## Conclusion

Our study accentuated the beneficial effects of bioflavonoid derivative TX by analyzing differential gene expression in blood cells. Transcriptomic analysis demonstrated that the expression of fifteen genes was significantly changed by the treatment with TX, among which 5 genes were up-regulated and 10 genes were down-regulated. DAG analysis showed that these genes could influence T cell-mediated cytotoxicity (q = 0.000149), telencephalon development (q = 0.000374), cell membrane (q = 5.04e-06), endoplasmic reticulum exports (q = 0.000153), β2-microglobule binding (q = 0.000278), TAP-binding (q = 0.000165), and T cell and neuropeptide Y receptor binding.

Among the 15 DEGs, TX has neuroprotective effects and improves cognitive impairment by up-regulating *TN-R* expression, anti-inflammatory effects and inhibits diabetes by down-regulating *Npy* and *Fcnb*, anti-cancer effects by changing ribosome protein gene rps2, effects on cardiovascular disease by up-regulating *Rpl17* and down-regulating robo1, and anti-oxidative activities by changing *Rpl3 and Hbq1a* expression. In addition, *H2-k1* and *Pnp2* expression was also changed. They are involved in positive regulation of T cell-mediated cytotoxicity and T cell proliferation. *Srp14* and *Arhgdig* expression were also change, which affect synthesis and export of proteins.

## Supporting information

S1 Fig Distribution of sample expression. (PDF)

S2 Fig Counts of differentially expressed genes (DEGs). (PDF)

S1 Table Exon_mapping. (XLS)

S2 Table Filter. (XLS)

S3 Table Mapping results. (XLS)

S4 Table rpkm log2 values. (XLS)

S5 Table PCR results. (XLS)
