# Supplementary figures and images for: Transcriptomic analysis of gene expression in mice treated with troxerutin
Source: PLoS One. 2017 Nov 30;12(11):e0188261. doi: 10.1371/journal.pone.0188261 (PMC5708793; doi:10.1371/journal.pone.0188261)

# Distrubution of Sample Expression

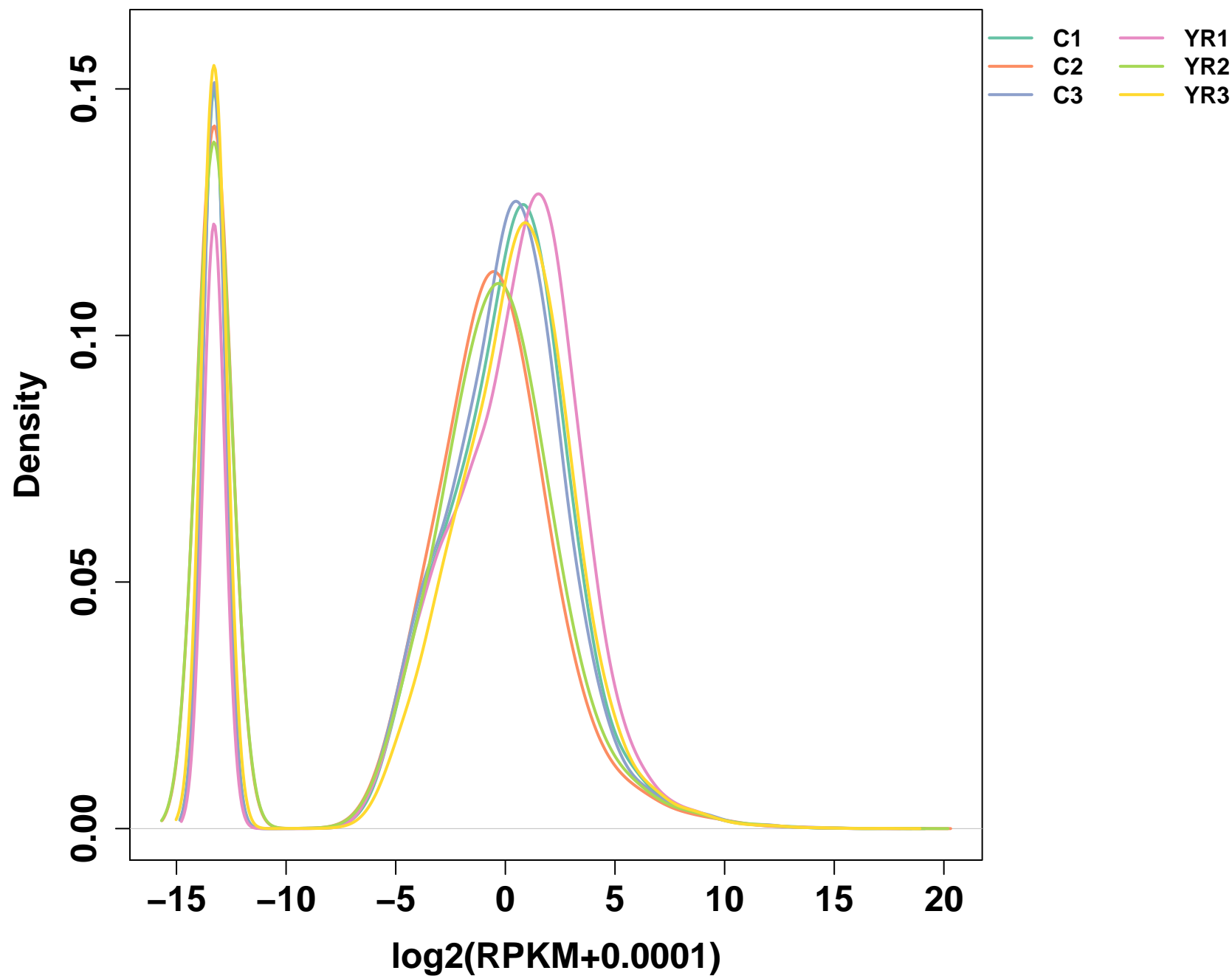

Supplement: S1 Fig — (PDF) [file pone.0188261.s001.pdf]

# DE Gene Count

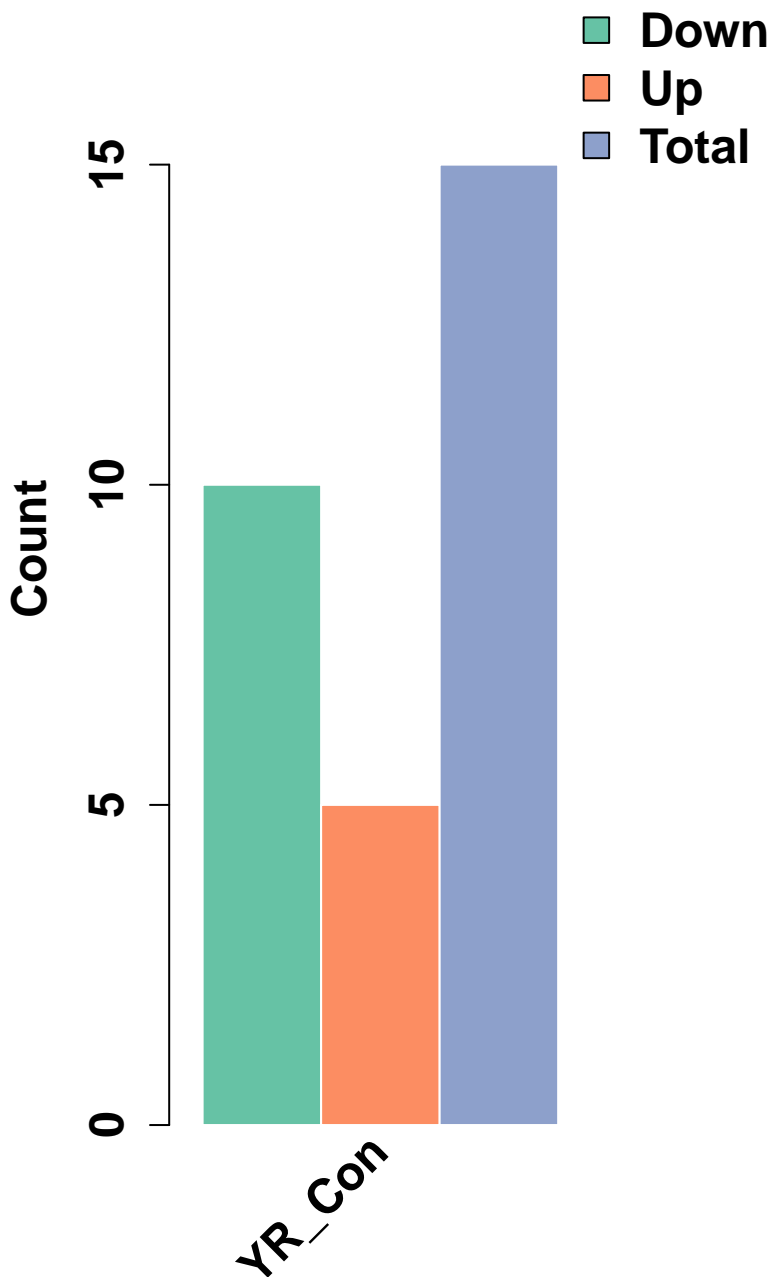

Supplement: S2 Fig — (PDF) [file pone.0188261.s002.pdf]
